# Cranioplasty after Two Giant Intraosseous Angiolipomas of the Cranium: Case Report and Literature Review

**DOI:** 10.3390/healthcare10040655

**Published:** 2022-03-31

**Authors:** Aurel George Mohan, Alexandru Vlad Ciurea, Iulian Antoniac, Veronica Manescu (Paltanea), Alin Bodog, Octavian Maghiar, Lavinia Marcut, Adrian Ghiurau, Florian Bodog

**Affiliations:** 1 Department of Neurosurgery, Clinical Emergency Hospital Oradea, 65 Gheorghe Doja Street, RO-410169 Oradea, Romania; mohanaurel@yahoo.com (A.G.M.); ghiurau.adrian@yahoo.com (A.G.); 2 Faculty of Medicine and Pharmacy, University of Oradea, 10 P-ta 1 December Street, RO-410073 Oradea, Romania; octimaghiar@yahoo.com (O.M.); rat_lavinia@yahoo.com (L.M.); fbodog@gmail.com (F.B.); 3 Faculty of Medicine, University of Medicine and Pharmacy “Carol Davila”, Dionisie Lupu Street, No. 37, RO-4192910 Bucharest, Romania; prof.avciurea@gmail.com; 4 Academy of Romania Scientists, 54 Splaiul Independentei, RO-050094 Bucharest, Romania; antoniac.iulian@gmail.com; 5 Faculty of Material Science and Engineering, University Politehnica of Bucharest, 313 Splaiul Independentei, District 6, RO-060042 Bucharest, Romania; 6 Faculty of Electrical Engineering, University Politehnica of Bucharest, 313 Splaiul Independentei, District 6, RO-060042 Bucharest, Romania; 7 Intensive Care Unit, Clinical Emergency Hospital Oradea, 65 Gheorghe Doja Street, RO-410169 Oradea, Romania; 8 Department of Surgery, Faculty of Medicine and Pharmacy, University of Oradea, RO-410073 Oradea, Romania

**Keywords:** bone tumor, cranium, craniotomy, cranioplasty, intraosseous angiolipoma, rare disease, two stage surgical procedure

## Abstract

Angiolipomas are rare, benign tumors resulting from the proliferation of adipose tissue and blood vessels, most frequently encountered subcutaneously at the upper limbs and trunk level. Due to their rarity, few cases of intraosseous angiolipomas are presented in the literature. The paper reports a 50-year-old female case with intracranial hypertension syndrome, frontal and parietal headache, nausea, and vomiting symptoms increasing in intensity. A CT exam revealed two hypodense expansive intraosseous formations/lesions. The first one was located in the projection of the frontal bone and the second one was placed on the left parietal bone. After further investigations, a two-stage procedure was considered. A frontal craniotomy with excision of the intraosseous tumor was performed in the first stage. In the second stage, a left parietal craniotomy was done with excision of the intraosseous tumor combined with a cranioplasty procedure. The patient had a favorable postoperative evolution with no symptoms or neurological deficits. This is among the few reported cases of intraosseous angiolipoma located at the cranium level and the first case report of two intraosseous angiolipomas situated on the same site. The medical recommendation was a complete surgical excision of the lesion followed by cranioplasty.

## 1. Introduction

In the context of rare diseases, a case report represents a valuable resource that supports medical research and education [1,2]. Angiolipomas are benign, slow-growing tumors made of proliferated adipose tissue and blood vessels. Usually, they appear subcutaneously, with the upper limbs and trunk being the most common sites [3,4].

Intraosseous angiolipomas (IOALs) are among the rarest benign bone tumors, first described by Polte et al. in 1976 [5]. With an incidence of 0.1% of all bone tumors, they generally occur in long bones (tibia, humerus, ribs) and present few clinical manifestations consisting of local tenderness and pain. It is important to note that even though we are referring to a benign tumor, it can cause fat necrosis, dystrophic calcification, and cyst formation leading to the destruction of bone tissue. Intraosseous angiolipomas occurring at the skull level are rare. Most common cases of intraosseous angiolipomas are situated in the ribs and jaw, with only a few cases being reported at the skull level according to the literature [6,7]. The disease often presents with an asymptomatic form; therefore, the actual incidence of the disease might be higher than current statistics [8,9]. Based on X-ray images, intraosseous angiolipomas are present in the marrow space and expand the bone. Computer tomography investigations show that these lesions are characterized by an essential fatty component, exhibiting septations and bony trabeculae. On T1 magnetic resonance imaging (MRI), intraosseous angiolipomas appear to be hyperintense, and T2 imaging also puts in evidence flow-voids that are characteristic of high vascularity tissues.

## 2. Literature Review about Intraosseous Angiolipomas (IOALs)

There are only a few cases of IOALs reported in the literature and even fewer IOALs of the cranium.

We performed a systematic review of the literature in two of the most prestigious databases (PubMed and Web of Science) using an advanced search function on 1 March 2022. The search was conducted using Boolean search operators and MeSH terms: “((intraosseous angiolipoma) AND ((skull) OR (cranium)))”. PubMed returned seven articles and Web of Science four articles using this method. One article was removed from the PubMed search results since it presented a lipoma and not an angiolipoma of the skull. All articles from Web of Science can be found in PubMed databases. Including our case presentation, only seven cases of intraosseous angiolipoma of the skull are described in the literature. The clinical cases description is summarized in Table 1.

Yu et al. [7] reported the case of a 50-year-old man who presented at the clinic with right parietal swelling of 3 cm. He first refused the surgical intervention, but after 11 years of follow-up, complete surgical excision of the intraosseous lesion was performed. The CT scan evidenced the lesion expansion in the right parietal bone, exhibiting bony spicules from the inner and outer plates. The cerebral angiography showed a distinct hypervascular appearance. The blood supply was predominantly from the right meningeal artery, with a minor contribution from superficial temporal and occipital arteries. A two-stage procedure was applied composed of total excision of the bone, where the lesion was located, and a titanium cranioplasty. The excised bone was investigated through histopathology, and the surgical margins were put in evidence. Patient bleeding was easy to control, and the man was symptom-free with no neurological alteration after the surgical treatment. The histopathology analysis showed a nonencapsulated lesion, mainly containing mature, highly vascularized adipose tissue. Fibrous connective aggregates of scattered mast cells and macrophages were put in evidence. The residual bone was characterized by thin trabeculae that contained osteocytes. The thrombosis was absent. This case was one of the few presented in the literature, and through a total resection of the tumor, the patient was free of disease, with no neurological damage.

Another interesting case of IOAL was described by Nguyen et al. [6]. In their paper, a 55-year-old-man presented at the clinic with symptoms of headache, vomiting, nausea, and double vision due to an intraosseous lesion located in the right frontal part of the cranium. The patient’s medical history of hypertension, diabetes, and smoking for more than 10 years was associated with an invasive ductal breast carcinoma. During the physical examination, it was concluded that the cranial nerves were in good condition. A CT scan without contrast substance evidenced a lesion of 4.3 × 2.2 × 1.7 cm^3^ located in the right frontal calvarial part, including trabeculated bone and fatty components with no invasion of the soft tissue and brain parenchyma. The surgical treatment for angiolipoma was divided into two steps. Firstly, an image-guided craniectomy was done to cut the calvarial tumor. After that, a titanium mesh cranioplasty was applied. No residual tumor was reported, and after postoperative day 2, the patient’s headache was improved. The histopathological investigation showed that the adipose part was composed through normal lobules, separated by thin fibrous septa.

Atilgan et al. [9] presented an intraosseous angiolipoma placed in the frontal bone of a 16-year-old female patient. Her symptoms were the right side of the frontal bone swelling and headache. Using CT scan tomography, a clearly contoured hypodense-lytic lesion with a 2 cm diameter was evidenced. Soft tissue inclusions or bone damages were not present. A frontal craniectomy and cranioplasty treatment was used. A disk-shaped bone with a diameter of 2.5 cm and a cavity placed in the middle of the bone were noticed. This bone was sectioned, and a yellow-brown lesion was put in evidence. The lesion exhibited bone trabeculae infiltration, cortical bone erosion, mature adipose tissue, and blood vessels. Numerous mast cells were noticed in the lesion, and there were no signs of necrosis or mitosis. The lesion was successfully identified as an angiolipoma and after a 1-year follow-up, no complications or recurrence was observed.

A giant intradiploic angiolipoma of the skull was reported by Amirjamshidi et al. [10]. A 41-year-old female patient presenting a prominent bulging on the right parietal region was investigated with the help of CT scans and MRI imaging techniques. The woman suffered from headaches for about 4 months. She had no nausea or epilepsy attacks. After physical examination, it was concluded that nerves were intact, no hemiparesis or papilledema was detected. CT images, done without contrast substance, put in evidence a densely trabeculated lesion, which contained calcified elements. MRI showed a sizeable intraosseous right frontotemporoparietooccipital lesion. It exhibited a mass effect on the patient’s skull. All the investigations put in evidence a non-homogenous structure of the tumor. A big skin incision was done, and it was noticed that the lesion did not infiltrate the skin, but the periosteum was not intact. There were no observed infiltrations of the tumor to the adjacent dural layer. A titanium mesh was used to repair the bone defect after the surgery. Histopathology showed a mixed structure with trabeculae bone and adipose tissue with small blood vessels that contained fibrin thrombi. After 23 months, the patient did not present tumor recurrence.

Singh et al. [8] reported a giant calvarial intraosseous angiolipoma in the case of a 30-year-old female. The lesion was present 5 years ago, and it enlarged after the patient pregnancy. On physical examination, a good and healthy overall appearance of the woman was noticed, with no signs of weakness or numbness in her extremities and no difficulties in moving her extremities. She did not exhibit visual abnormalities or seizures. She reported only an altered sensation over her right parietal region. The pain was not a present symptom, and her cranial nerves were in good condition. Overall, she was in good health, and her only problems were pulmonary artery stenosis and irritable bowel syndrome. CT scans put in evidence a 6.4 × 6.4 × 4 cm^3^ right parietal calvarial mass. MRI showed a lesion placed at the right parietal bone center with an extension to the cranial vertex. During the surgery, it was noticed that the tumor was extradural, and it had developed into a cranial cavity, with a biconvex shape. In order to fix the osseous defect, a Synthes PEEK implant was used. In this case, histopathology evidenced mature adipose tissue with thin blood vessels. After the surgery, the patient recovered well, and no signs of cosmetic deformities were noticed.

Morgan et al. [11] reported in 2020 a case of angiolipoma. In this paper, a 61-year-old female with a history of squamous and basal cell carcinomas, arthritis, and hypertension presented to the clinic for a palpable left frontoparietal bone lesion. Six months before, she had suffered a trauma. CT and MRI revealed the lesion’s existence. A two-stage surgical treatment was applied. A left frontoparietal craniectomy followed by a cranioplasty were performed. The histopathology analysis put in evidence and underlined the diagnosis of angiolipoma. After follow-up, the patient exhibited no recurrence of the tumor.

Our current study includes two intraosseous angiolipomas of the cranium, and according to our knowledge, this is the first case with two IOALs reported in the literature. We will highlight our findings and try to present the possible theories of the lesion genesis according to the above-described literature review. The patient diagnostic included the MRI and CT scan investigations and immunohistochemical characteristics.

## 3. Case Presentation

We present the case of a 50-year-old female patient that came to the Emergency Department with intracranial hypertension syndrome, accusing frontal and parietal headache, nausea, and vomiting symptoms from a month before, increasing in intensity.

After CT cranial exam, two expansive intraosseous formations were found: one in the projection of the frontal bone measuring 80 × 34 mm^2^ and another on the left parietal level measuring 70 × 21 mm^2^. Both lesions were present with a biconvex imaging appearance, hypodensity, multiple interior calcifications, and a marked thinning of the inner and outer plates, exerting a compressive effect on the cerebral parenchyma.

**Figure 1:**
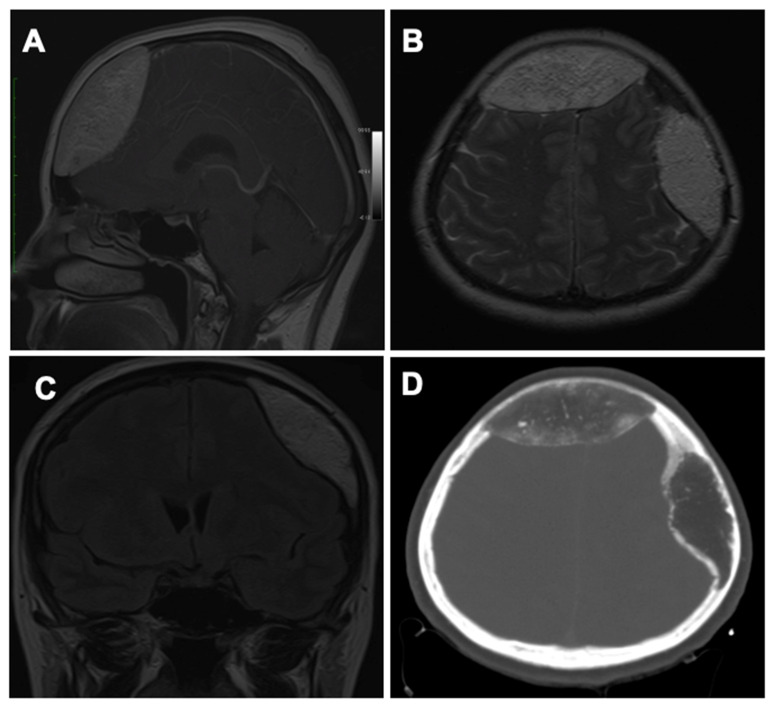
Preoperative neuroimaging: (**A**) Sagittal MRI scan showing the frontal lesion. (**B**) Axial MRI scan showing both the frontal and the parietal lesions. (**C**) Coronal MRI scan showing the parietal lesion. (**D**) Axial CT scan highlighting the hypodense aspect of frontal and parietal osseous lesions.

The patient was admitted to the Neurosurgery Clinic for specialized treatment. Under depletive treatment, the symptoms of intracranial hypertension were remitted. A cranial MRI scan confirmed the nature of the compressive extra-axial left frontal and parietal intraosseous lesions on the cerebral parenchyma, as seen in Figure 1. T1 and T2 sequences were performed with medium contrast.

The patient consented to a two-stage procedure consisting of a craniotomy with total excision of the frontal intraosseous tumor in the first stage, followed by a second craniectomy in the left parietal region with total excision of the intraosseous tumor and a titanium cranioplasty intervention. In the first stage, surgery was performed on the frontal lesion. A bilateral frontal craniotomy with excision of the intraosseous tumor and the reconstruction of the area using the intact external plate was performed.

**Figure 2:**
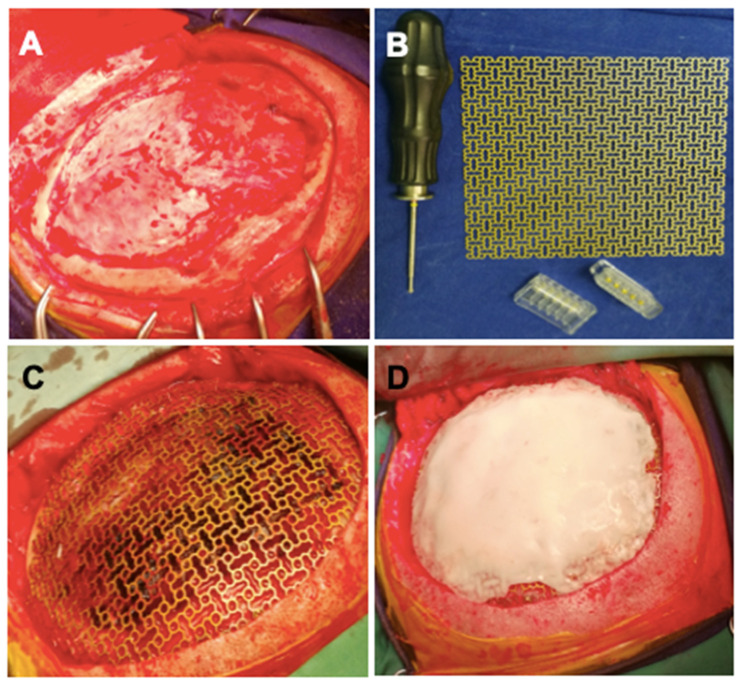
(**A**) Left frontoparietal craniotomy. (**B**) Titanium mesh with titanium monoaxial screws clamping system. (**C**) The aspect of applied titanium mesh. (**D**) PMMA cementation over titanium mesh.

**Figure 3:**
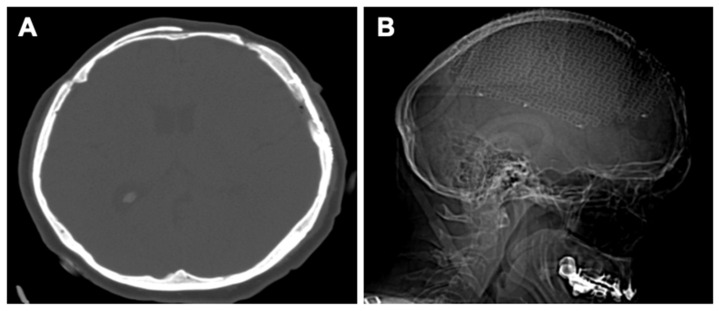
Postoperative neuroimaging: (**A**) Axial CT scan. (**B**) Sagittal radiographic scan.

In the second stage, 6 weeks later, surgery was performed for the second lesion located in the left parietal region. A left frontoparietal craniectomy was performed with complete ablation of the lesion. A titanium mesh with self-tapping titanium screws adapted for the bone defect was used for the reconstruction of the skull. Due to the large size of the bone defect, it was decided to strengthen the titanium mesh by applying a polymethylmethacrylate (PMMA) cover. Clinical images of the procedure are represented in Figure 2, and postoperative neuroimaging is presented in Figure 3.

**Figure 4:**
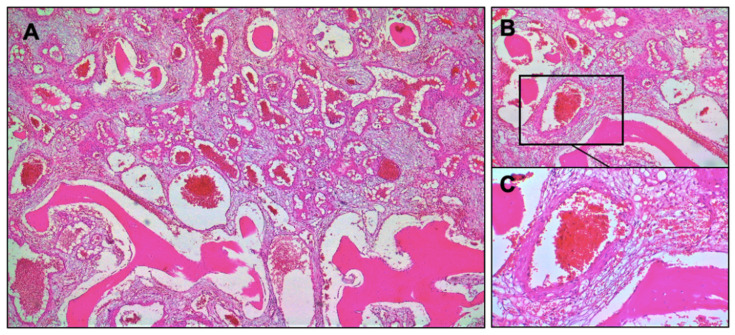
Microscopical images related to the histological investigation—general aspects of the tissue, hematoxylin and eosin (H&E) section: (**A**) Tumoral proliferation formed of venous blood vessels and trabecular bone tissue (H&E, ×4 original magnification); (**B**) bone lamellae replaced by proliferation of blood vessels in a myxoid matrix (H&E, ×10 original magnification); (**C**) bone lamellae that present a row of osteoblasts on the edge and a sanguine vessel of venous type disposed in a myxoid matrix (H&E, ×20 original magnification).

Specimens were cut and stained with hematoxylin and eosin (H&E) and Masson’s trichrome. Histological evaluation revealed a mixture of mature adipose tissue and thin-walled blood vessels. A general aspect of the tissue can be observed in Figure 4, in which is highlighted the tumoral proliferation formed of venous blood vessels with variable calibers and trabecular bone tissue.

**Figure 5:**
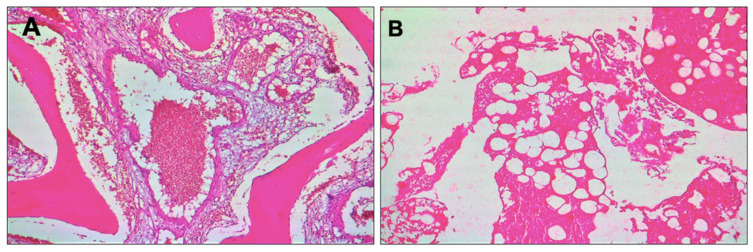
Microscopical images related to some details obtained during the histological investigation—hematoxylin and eosin (H&E) section: (**A**) Dilated blood vessel that replace the hematogenous marrow (×10 original magnification); (**B**) areas with hemorrhagic necrosis located in an angiolipomatous formation (H&E, ×4 original magnification).

The microscopic examination presented in Figure 5 revealed bone lamellae replaced by a proliferation of blood vessels without a well delimited internal elastic membrane, blood vessels that replace the hematogenous marrow and hemorrhagic necrosis areas.

**Figure 6:**
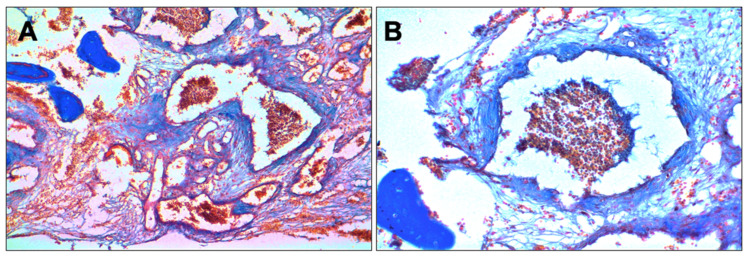
Microscopical images related to the proliferation of blood vessels obtained during the histological investigation—Masson’s trichrome section: (**A**) Blood vessels proliferation in trabecular bone tissue (×4 original magnification); (**B**) dilated venous blood vessel with red blood cells (RBCs) present in the lumen (×10 original magnification).

Figure 6 presents the proliferation of blood vessels in a Masson’s trichrome section.

The patient presented a favorable postoperative evolution with no symptoms and neurological deficits. The cerebral volumetry and the aesthetic aspect were restored. The patient was seen at regular time intervals of 1-, 6-, 12-, and 24-months remaining symptom-free with no recurrence after a follow-up of 2 years.

## 4. Discussion

The case described herein involves two expansive intraosseous angiolipomas of the cranium, one located in the frontal bone and the other in the left-parietal bone. The lesions presented a hypodense aspect with multiple interior calcifications and a marked thinning of the inner and outer plates. Further MRI investigations confirmed the expansive characteristic of intraosseous lesions that exerted a compressive effect on the cerebral parenchyma. Given the presence of two extended IOALs of the cranium, a two-stage procedure was considered.

Angiolipomas represent tumoral proliferations formed of various blood vessels (arteries, veins, capillaries) and trabecular bone tissue, with areas of hemorrhagic necrosis sometimes present. The term angiolipoma was firstly described by Howard and Helwig in 1960 [12]. Angiolipomas have a clinical appearance in the form of different size nodules situated at the subcutaneous level, and besides being slightly firmer, they can easily be mistaken as lipomas [13]. They also present a variable ratio of adipose to vascular tissue, but in comparison with lipomas, the vascular component represents a more prominent feature. Intraosseous angiolipomas (IOALs) are the rarest benign bone tumors, often occurring in long bones. Preoperative diagnosis of an IOAL may be difficult as it shares neuroimaging features with different other adipose neoplasms affecting bone tissue such as lipomas [14], fibrous dysplasia [15,16], angiomas, and meningiomas [6,17].

Currently, modern imaging techniques manage to narrow the differential diagnosis and offer better insight [18]. A CT scan of intraosseous angiolipoma indicates a hypodense lesion in the form of an expanding skull mass involving the entire thickness of the cranium. Thus, a correlation of the neuroimaging features (CT and MRI scans, radiographic) with the clinical and histologic characteristics can help identify the common features of IOALs and determine proper management of the intervention [10,19]. For intraosseous angiolipomas with highly affected bone tissue, biomaterials such as titanium, polyether ether ketone (PEEK), and polymethyl methacrylate (PMMA) with the help of 3D printing technology can be used in the cranioplasty procedure [20].

As we have investigated in the literature, just a few reported cases of IOALs of the cranium were reported (Table 1). Summarizing the systematic and clinical review presented in Section 2, 5 patients (71.4%) were female and 2 patients (28.6%) male. The average diagnosis age was 41.85 years, the youngest patient being a minor (16 years) and the eldest being 61 years old. Regarding localization, 5 cases (71.4%) were situated on the right side, affecting the frontal and/or the parietal bone equally. A total of 4 cases (57.1%) had just one cranial bone affected, 2 cases (28.5%) had two cranial bones affected, and 1 case (14.2%) had 4 cranial bones affected (right frontotemporoparietooccipital lesion). Symptoms were nonspecific, headache being the most common (4 cases; 57.1%), but it was absent in 2 cases (28.5%). All cases were investigated using CT scans and 5 cases (71.4%) used an MRI scan in addition. The average lesion size was 7.38 cm, the largest being 20 cm and the smallest 2 cm. Mass effect was present in 6 cases (85.7%). All cases were surgically managed with complete en-bloc resection of the lesion, followed by cranioplasty. No case presented postoperative complications or recurrence of the tumor. All cases had a great recovery, with remissions of the symptoms where they were present.

## 5. Conclusions

We report a case of two rare intraosseous angiolipomas of the cranium situated in the frontal and parietal bone, the first such case documented and among the few intraosseous angiolipoma of the cranium reported in the literature. It is important to understand the extent of this disease and how to perform a differential diagnosis and manage the intervention properly. We recommend the main treatment for intraosseous angiolipomas, which consists of complete surgical excision of the lesion. It should be noted that even benign tumors can affect the bone up to destruction and create multiple abnormalities.

## Figures and Tables

**Table 1:** Summary of cases of intraosseous angiolipoma of the cranium.

Case	Age/Sex	Location	Signs/Symptoms	Other	Radiographic Investigations	Treatment
Yu et al., 2009 [7]	50/M	Right parietal bone	Asymptomatic	Swelling increasing in size; minor trauma history	CT and angiography: 7 cm focal hypodense lesion with bony spicules associated with hypervascularity and mass effect on the parietal lobe	En-bloc resection of lesion with titanium cranioplasty
Nguyen et al., 2014 [6]	55/M	Right frontal bone	Headache, nausea, vomiting, and double vision	>10 pack-year history of cigarette smoking; also found to have invasive ductal breast carcinoma	CT and MRI: 4.3 cm heterogeneously mixture of trabeculated bone and fatty components with mild mass effect on the adjacent frontal lobe	En-bloc resection of lesion with titanium cranioplasty
Atilgan et al., 2014 [9]	16/F	Right frontal bone	Scalp swelling and headache	No history of traumatic episodes	CT: 2 cm well-defined hypodense-lytic lesion to the right of the frontal lobe	En-bloc resection of lesion with titanium cranioplasty
Amirjamshidi et al., 2014 [10]	41/F	Right frontoparietotemporal bone and part of the occipital bone	Mild headache of about 4 months duration without nausea or vomiting	Lesion grown during the previous 2 years with recently tenderness	CT and MRI: 20 cm densely intraosseous expansile lesion with calcified components and mass effect upon the adjacent dura	En-bloc resection of lesion with titanium cranioplasty
Singh et al., 2016 [8]	30/F	Right parietal bone	Altered sensation over the affected area without any pain with palpation	Lesion present 5 years prior to presentation; began to enlarge after pregnancy	CT and MRI: 6.4 cm calvarial mass with expansion and mass effect on the right parietal lobe	En-bloc resection of lesion with PEEK cranioplasty
Morgan et al. [11], 2020	61/F	Left frontoparietal bone	Asymptomatic	Swelling; minor head trauma 6 months before; history of squamous cell carcinoma and basal cell carcinoma	CT and MRI: 4.4 cm expansile, spiculated, enhancing lesion with superficial soft tissue component and cerebral compression	En-bloc resection of lesion with cranioplasty
Current study	50/F	Frontal bone and left parietal bone	Frontal and parietal headache, nausea, and vomiting	Symptoms increasing in intensity from a month before	CT and MRI: two hypodense lesions (7 cm and 8 cm) with multiple interior calcifications and compressive effect on the cerebral parenchyma	En-bloc resection in two stages: 1. Frontal craniotomy and reconstruction with intact external plate; 2. Left parietal craniotomy with titanium and PMMA cranioplasty

## Data Availability

Not applicable.
